# Development and Validation of a Random Forest Diagnostic Model of Acute Myocardial Infarction Based on Ferroptosis-Related Genes in Circulating Endothelial Cells

**DOI:** 10.3389/fcvm.2021.663509

**Published:** 2021-06-28

**Authors:** Chen Yifan, Shi Jianfeng, Pu Jun

**Affiliations:** State Key Laboratory for Oncogenes and Related Genes, Division of Cardiology, Renji Hospital, School of Medicine, Shanghai Jiao Tong University, Shanghai Cancer Institute, Shanghai, China

**Keywords:** ferroptosis, myocardial infarction, diagnostic model, random forest, supervised machine learning

## Abstract

The high incidence and mortality of acute myocardial infarction (MI) drastically threaten human life and health. In the past few decades, the rise of reperfusion therapy has significantly reduced the mortality rate, but the MI diagnosis is still by means of the identification of myocardial injury markers without highly specific biomarkers of microcirculation disorders. Ferroptosis is a novel reported type of programmed cell death, which plays an important role in cancer development. Maintaining iron homeostasis in cells is essential for heart function, and its role in the pathological process of ischemic organ damages remains unclear. Being quickly detected through blood tests, circulating endothelial cells (CECs) have the potential for early judgment of early microcirculation disorders. In order to explore the role of ferroptosis-related genes in the early diagnosis of acute MI, we relied on two data sets from the GEO database to first detect eight ferroptosis-related genes differentially expressed in CECs between the MI and healthy groups in this study. After comparing different supervised learning algorithms, we constructed a random forest diagnosis model for acute MI based on these ferroptosis-related genes with a compelling diagnostic performance in both the validation (AUC = 0.8550) and test set (AUC = 0.7308), respectively. These results suggest that the ferroptosis-related genes might play an important role in the early stage of MI and have the potential as specific diagnostic biomarkers for MI.

## Introduction

Myocardial infarction (MI), the most common and most precarious outcome of coronary heart disease, endangers the health of the majority (1). With the progress of interventional and reperfusion therapy in recent years, the mortality rate of acute MI has been significantly reduced. However, it cannot be ignored that there is still a lack of efficient tools and biomarkers for the early diagnosis of acute MI. Even in the early stage of acute MI, every hour of early diagnosis and timely treatment could increase the survival rate by about 15% (2, 3). Specific markers of myocardial injury, such as cardiac troponin T (cTnT) (4) and typical changes on an electrocardiogram (ECG) (5) take the top priority for MI diagnosis in recent clinical guidelines (6). However, such diagnostic strategy still faces a lot of challenges. The cTnT in myocardial cells lacks timeliness for the early diagnosis of acute MI because it only reflects myocardial damage and even rupture caused by ischemia, hypoxia, and other factors without characterizing the early myocardial perfusion abnormalities. Moreover, the half-life of cTnT in the blood is too long to identify the reinfarction (7). What is more, typical changes on ECG of MI are not always stable and could be interfered with by other cardiomyopathy. Hence, measures should be taken immediately to explore novel biomarkers for early diagnosis.

Circulating endothelial cells (CECs) are derived from the metabolism of the vascular endothelium (8), which directly reflects the contractile function of blood vessels, the perfusion of capillaries, and the state of ischemia and hypoxia earlier than cardiomyocytes (9). Meanwhile, ischemia and hypoxia directly lead to abnormal metabolism and programmed death of vascular endothelial cells, which could also be obtained from the state of circulating endothelial cells through direct blood tests.

Ferroptosis is a newly discovered type of programmed cell death in recent years. It is well-known for its iron-dependent phospholipid peroxidation process to cause cell membrane damage and even cell death (10). In fact, iron metabolism is tightly regulated in the organism, and excessive Fe^2+^ could induce the production of active reactive oxygen species (ROS), which would trigger the oxidative stress. Meanwhile, glutathione peroxidase (GPX4) could reverse lipid peroxidation and ferroptosis by consuming glutathione. Recent studies show that the regulation of ferroptosis is associated with autophagy in cancer (11). And CD8^+^ T cells activated by immunotherapy can exert their antitumor effects by enhancing the ferroptosis of tumor cells. Although this evidence demonstrates the importance of ferroptosis in cancer, few studies focus on its role in ischemic disease. In the field of cerebrovascular disease, it is reported that activating the expression of GPX4 could protect neurons in the ischemic stroke model (12). However, the underlying regulation of ferroptosis is still in the veil in the field of cardiovascular disease, especially in MI (13).

Here, we screened the differentially expressed genes in CECs of acute MI patients from the GEO database. Then, the ferroptosis-related genes collected from the FerrDb database (http://zhounan.org/ferrdb) (14) and other previous literature (15–18) were utilized to identify the differential ferroptosis-related genes in acute MI. Finally, we established and evaluated a random forest diagnostic model based on these genes and verified it in another data set in GEO after comparing three different supervised machine learning algorithms.

## Methods and Materials

### Original Gene Expression Profiles Acquisition and Data Preprocessing

Two related CEC databases, GSE66360 (19) and GSE48060 (20), were selected and downloaded from the GEO database (https://www.ncbi.nlm.nih.gov/GEO/). Both of these data sets were updated on March 25, 2019. Although these two databases shared the same supplementary microarray probe platform GPL570, GSE66360 alone was selected as the training and validation sets. Meanwhile, GSE66360 is confirmed to be matched by both gender and age in the experimental and control groups (19). GSE48060 was treated as the test set to avoid the possible interference of a batch effect. Then, quality control and normalization of these two gene expression profiles were conducted through the scale function in R 4.0.3 software.

### Differential Gene Expression

The latest version of the “stringr” and “limma” packages in R 4.0.3 software were used to perform differential expression analysis. The fold change (FC) was calculated based on the average gene expression of the acute MI and control groups, and the differentially expressed genes were defined by the cutoff values (FC > 1.5 and *P* < 0.05). Meanwhile, the gene probe IDs were matched with the “Gene Symbol” through “Gene ID Conversion” in the DAVID online database (http://david.ncifcrf.gov/) (21).

### Functional Enrichment Analysis of Differential Genes

The DAVID online database was also adopted for the gene ontology (GO) enrichment analysis, including three aspects of biological process (BP), cell composition (CC), and molecular function (MF) for functional annotation (21). Meanwhile, the KEGG online database (http://genome.jp/kegg/pathway.html) (22) was used to analyze the KEGG signal pathway of those differentially expressed genes. In addition, hierarchical clustering of samples and differential genes were performed and visualized through the “heatmap” R package.

### Collection of Ferroptosis-Related Genes

Ferroptosis-related genes were collected and retrieved from the FerrDb database (http://zhounan.org/ferrdb) (14), and other previous literature (15–18) was referenced for proofreading and completion. All the ferroptosis-related genes are provided in Supplementary Table 1.

### Analysis of Differential Ferroptosis-Related Genes

The latest version of the “venneuler” package in R 4.0.3 software was applied to depict the intersection of differential and ferroptosis-related genes, and the “seaborn” library in Python 3.90 was used to visualize the expression of different ferroptosis-related genes between the MI and control groups, and Student's *t*-test was adopted as the statistical analysis by “scipy.stats” Python library. The *P*-value < 0.05 was considered statistically significant, and all *P*-values were two-tailed. Meanwhile, the STRING database (http://string-db.org/) (23) was used to perform a protein–protein interaction (PPI) network on the differentially expressed proteins of those ferroptosis-related genes. In addition, principal component analysis (PCA) was performed on the differentially expressed ferroptosis-related genes as a dimension-reduction strategy to distinguish the MI and control groups through the “sklearn” Python library.

### Construction of Diagnostic Prediction Model Through Machine Learning

Differential expressions of ferroptosis-related genes were treated as independent variables to construct a prediction model for the diagnosis of acute MI based on CECs. In this study, we used the GSE66360 data set as the training and validation set (4:1), and the GSE48060 data set was treated as the test set. In order to prevent the overfitting phenomenon caused by the complex model, K-fold cross-validation with cv = 15 was adopted in this study to improve the generalization ability of the training set. Feature selection was implemented through the “sklearn.model_selection” Python library. Then, three different supervised machine learning algorithms were used to initially explore the diagnostic prediction model. The logistic regression, support vector classification, and random forest models were, respectively, built through the “sklearn.linear_model,” “sklearn.svm,” and “sklearn.ensemble” Python libraries. After comparing the performance of different models, the random forest algorithm was selected, and some parameters and structures were adjusted to optimize this algorithm. ROC curves were visualized through the “matplotlib” Python library. Then, the validation set was used to verify the prediction model, and the test set was applied to demonstrate the generalization ability of this diagnostic model.

## Results

### Research Flow and the Collection of Ferroptosis-Related Genes

GSE66360, a CECs data set of acute MI with clinical information, was downloaded from the GEO database for further differential gene screening. This data set included 49 acute MI patients with strict diagnostic criteria and 50 healthy controls. Meanwhile, all the patient CECs were isolated from peripheral blood within 48 h of acute MI and identified with a CD146^+^ specific antigen. Then, differential expression and functional enrichment analyses were performed after quality control and normalization of the gene expression matrix.

**Figure 1:**
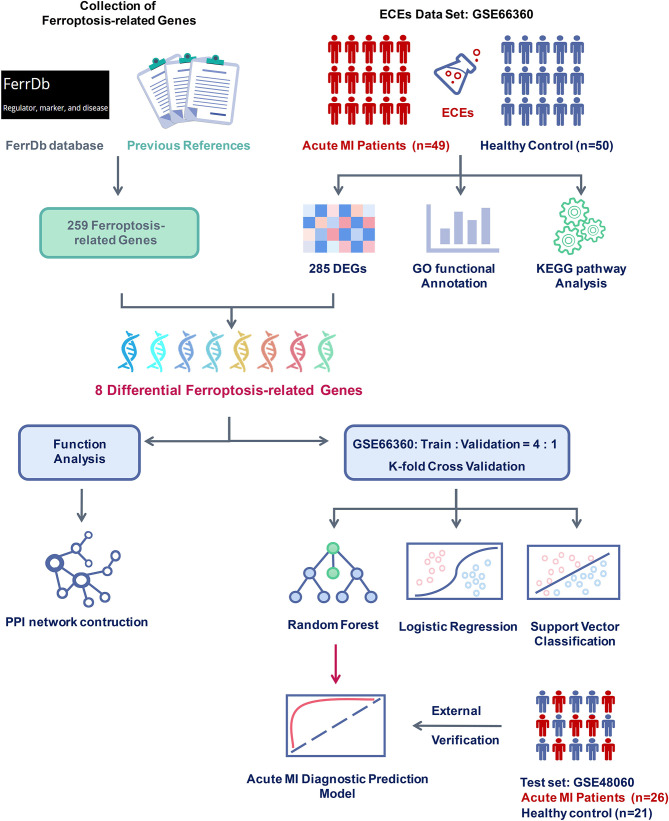
Flow chart of research design and analysis. GSE66360 was applied to analyze differentially expressed genes (DEGs) between acute myocardial infarction (MI) and healthy controls. Ferroptosis-related genes were collected from the FerrDb database and other previous references. After checking the DEGs and the ferroptosis-related genes, eight differential ferroptosis-related genes were selected to perform functional analysis and construct a clinical diagnosis model. Compared with different supervised learning algorithms, including logistic regression and support vector classification, the random forest algorithm was determined to build the acute MI diagnostic model and was confirmed with the external verification of GSE48060.

In addition, 259 ferroptosis-related genes were confirmed through the FerrDb database and other previous references after deduplication. Subsequently, the intersection of differentially expressed and ferroptosis-related genes were taken, and the expression differences of these genes were tested in the two groups. The PPI network of these differential ferroptosis-related genes was also built. Then, the GSE66360 data set was divided into the training and validation sets at a ratio of 4:1. Finally, a diagnostic prediction model of acute MI based on the random forest algorithm was constructed by those screened differential ferroptosis-related genes after K-fold cross-validation and algorithm comparison and verified on the test set GSE48060 including 26 acute MI patients with non-recurrent events and 21 normal controls (Figure 1).

### Verification and Functional Analysis of Differentially Expressed Genes in Acute MI

**Figure 2:**
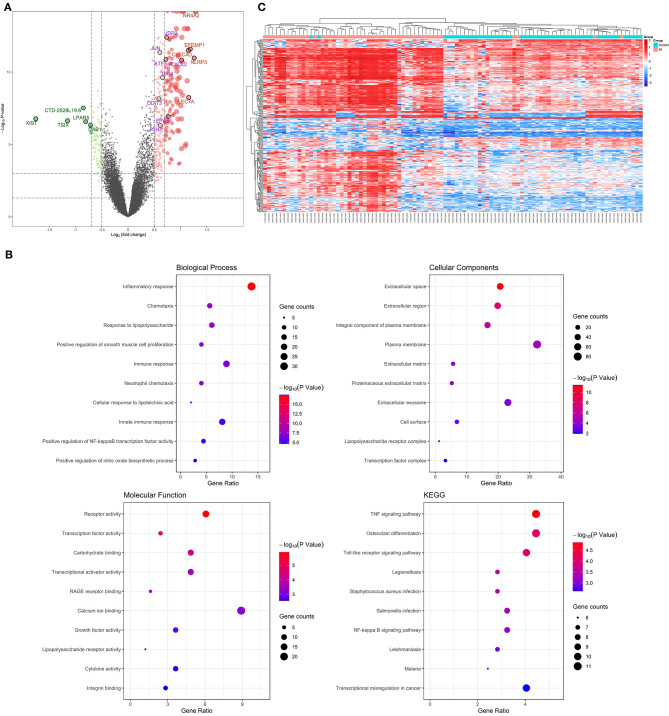
Verification and functional analysis of differentially expressed genes (DEGs) in acute myocardial infarction (MI). **(A)** DEGs in acute MI and control group by the (FC > 1.5 and *P* < 0.05) cutoff value (top five upregulated genes are marked in red; top five downregulated genes are marked in green; the following differential ferroptosis-related genes are marked in purple); **(B)** gene ontology (GO) enrichment analysis among these DEGs, including biological process (BP), cell composition (CC), and molecular function (MF), and the KEGG pathway enriched by these DEGs; **(C)** the hierarchical clustering of all the DEGs and clinical status.

The 99 samples in the MI and control groups of GSE66360 were normalized and the FC calculated through the “limma” R package. After setting the cutoff values (FC > 1.5 and *P* < 0.05), 256 differentially expressed genes of ECEs in the control and MI groups were screened, including 37 upregulated genes and 219 downregulated genes (Figure 2A). Meanwhile, the top five upregulated genes with huge significance were NR4A2, NLRP3, EFEMP1, CLEC7A, and CLEC4D in the MI group. The top five downregulated genes were XIST, TSIX, CTD-2528L19.6, LPAR5, and DAB1 in the MI group. Some of these genes were reported to be involved in various processes of the development of cardiovascular diseases, including hypoxia, autophagy, and oxidative stress (24–26).

In order to further explore the pathophysiology functions of these differentially expressed genes in acute MI, GO analysis according to the DAVID online database was adopted to cluster the BP, CC, and MF among them. The results were that most genes participated in the inflammatory response in BP, followed by extracellular space in CC and receptor activity in MF (Figure 2B).

In addition, the KEGG pathway analysis of these differentially expressed genes showed that the top three signal pathways with the largest number of enriched genes were the TNF signaling pathway, osteoclast differentiation, and Toll-like receptor signaling pathway (Figure 2B). All these pathway enrichments were also supported and echoed by corresponding literature (13, 27, 28).

What is more, hierarchical clustering was also applied to verify the reliability of these differential genes, and the two groups could be significantly distinguished according to a heat map (Figure 2C).

### Expression and Functional Analysis of Differential Ferroptosis-Related Genes

**Figure 3:**
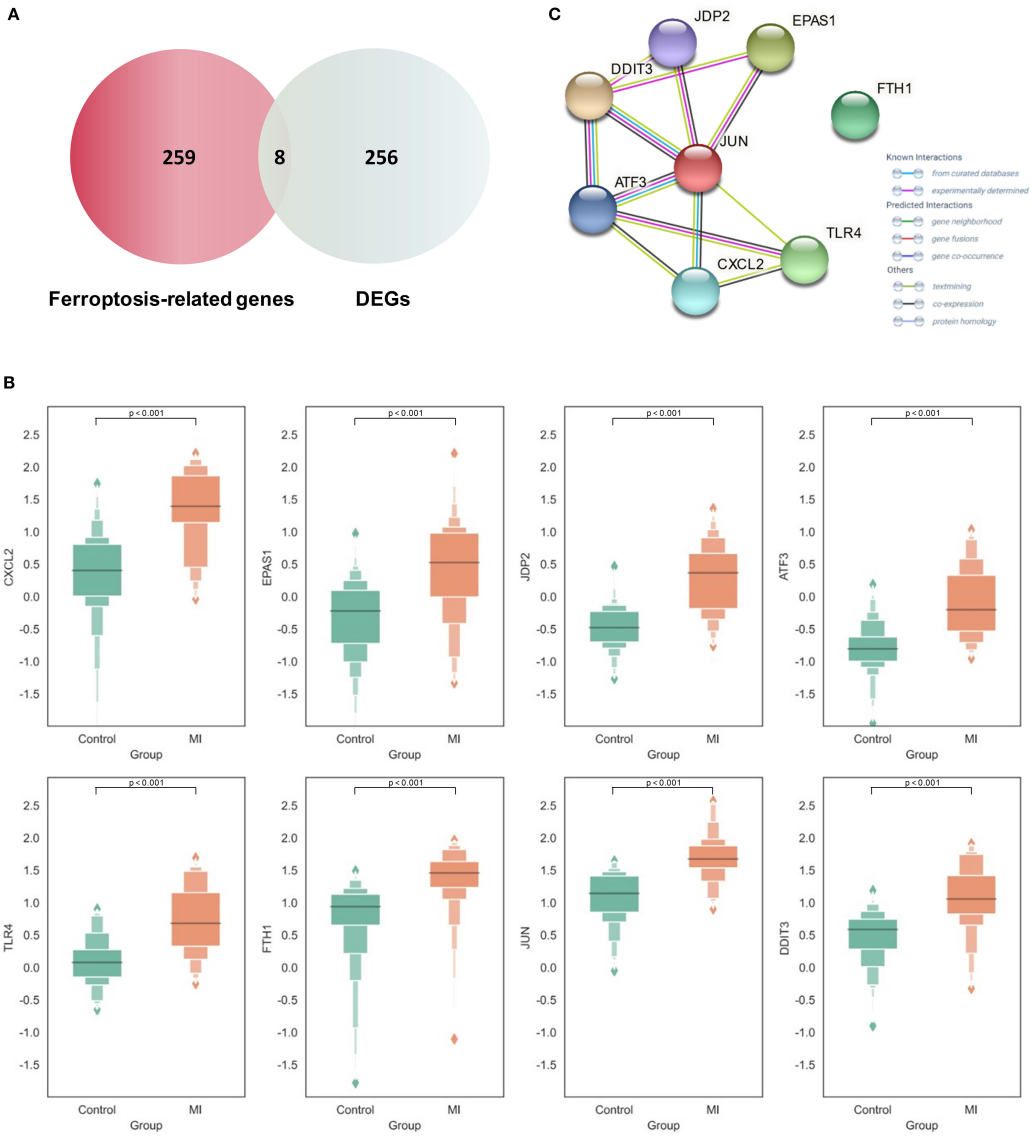
Expression and functional analysis of differential Ferroptosis-related genes in acute myocardial infarction (MI). **(A)** The intersection between the collected ferroptosis-related genes and differentially expressed genes (DEGs) in acute MI; **(B)** the expression of different ferroptosis-related genes between the acute MI and control groups with the two-tailed Student's *t*-test (*P* < 0.05 as significance); **(C)** the protein–protein interaction (PPI) network on those ferroptosis-related genes.

By intersecting the collected ferroptosis-related genes with the differentially expressed genes described above, eight differentially expressed ferroptosis-related genes including C-X-C motif chemokine ligand 2 (CXCL2), endothelial PAS domain protein 1 (EPAS1), Jun dimerization protein 2 (JDP2), activating transcription factor 3 (ATF3), Toll-like receptor 4 (TLR4), ferritin heavy chain 1 (FTH1), AP-1 transcription factor subunit (JUN), and DNA damage-inducible transcript 3 (DDIT3) were obtained (Figure 3A). The relevant information of all these genes is demonstrated in Table 1. All these differentially expressed ferroptosis-related genes were shown to be significantly highly expressed in the acute MI group with *P*-values < 0.0001 (Figure 3B).

**Table 1:** Summary of all these differential expressed ferroptosis-related genes in acute myocardial infarction (MI).

**Gene**	**Full name**	**Role in Ferroptosis**	**logFC**	***P*-value**
CXCL2	C-X-C motif chemokine ligand 2	Marker	1.019	1.51 × 10^−11^
EPAS1	Endothelial PAS domain protein 1	Driver	0.770	1.19 × 10^−7^
JDP2	Jun dimerization protein 2	Marker	0.736	4.39 × 10^−13^
ATF3	Activating transcription factor 3	Driver	0.722	1.33 × 10^−11^
TLR4	Toll-like receptor 4	Driver	0.659	2.46 × 10^−10^
FTH1	Ferritin heavy chain 1 (FTH1)	Marker	0.614	5 × 10^−7^
JUN	AP-1 transcription factor subunit	Suppressor	0.606	4.27 × 10^−12^
DDIT3	DNA damage-inducible transcript 3	Marker	0.588	7.1 × 10^−9^

Although the specific mechanism of ferroptosis in cardiovascular disease was not clear, our results first confirmed the potential role of these ferroptosis-related genes in acute MI. CXCL2 occupied the most obvious difference in expression, which was thought to be associated with neutrophil-mediated inflammation (29). However, it is also gradually recognized as a key factor involved in cellular ferroptotic response in recent years. Meanwhile, it is also shown that CXCL2 is significantly highly expressed in the plaques and peripheral blood mononuclear cells of patients with coronary atherosclerosis, and it might be closely related to the prognosis (30). Another significant difference was shown on EPAS1, which played a critical role in ferroptosis through lipid peroxidation. It could selectively enrich polyunsaturated lipids by upregulating hypoxia and lipid droplet-related protein. In some tumors, such as clear-cell carcinomas, EPAS1 may even promote the tumor-dependent ferroptotic death procession by recruiting some specific downstream factors (31). Another transcriptional regulator, JDP2, could activate the expression of inflammatory genes and promote fibrosis, which has been shown as a prognostic marker for MI patients to develop heart failure (32). As a key effector of ferroptosis, TLR4 plays an important role in reducing cardiomyocyte death and improving left ventricular remodeling. After knocking down TLR4, autophagy and ferroptosis could be alleviated through the TLR4 and NADPH oxidase 4 (NOX4) pathway, which provides a potential treatment strategy for heart failure (33).

At the same time, the STRING database was used to construct the PPI interaction network of these differential ferroptosis-related proteins (Figure 3C). It was revealed that JUN might be the hub node in all eight differential ferroptosis-related genes because it was related to almost all other genes except FTH1. In previous studies, Jun is shown to regulate the ferroptotic cell death with the help of hepatocyte nuclear factor 4 alpha (HNF4A) (16). Our results first report the potential role of Jun in acute MI by mediating abnormal ferroptosis. The following two key nodes are DDIT3 and ATF3, both of which are related to four other differential ferroptosis-related genes. The endoplasmic reticulum is an important organelle for maintaining cell homeostasis. As a key regulator of endoplasmic reticulum stress, DDIT3 is also reported to be involved in the ROS-dependent ferroptotic process (34), but its role in the pathological process of cardiovascular disease still remains unknown. In terms of ATF3, this famous common stress sensor could accelerate the progression of ferroptosis by inhibiting system Xc^−^ (35). Some studies also show that suppressing the expression of ATF3 could improve the prognosis of cardiovascular and cerebrovascular diseases through reducing cell death (36). Meanwhile, the isolation of FTH1 did not make sense. As a regulatory element of cellular iron storage, FTH1 is critical for maintaining intracellular iron homeostasis. Knockout of FTH1 is shown to induce ferroptosis through erastin, sorafenib, and other pathways in various disease models (37) while overexpression of FTH1 could restrain ferritinophagy to reduce ferroptosis (38). Moreover, FTH1-mediated iron metabolism disorder is shown to exacerbate myocardial damage during MI and reduce heart function (39).

### Establishment of the Diagnostic Model Based on Differential Ferroptosis-Related Genes

**Figure 4:**
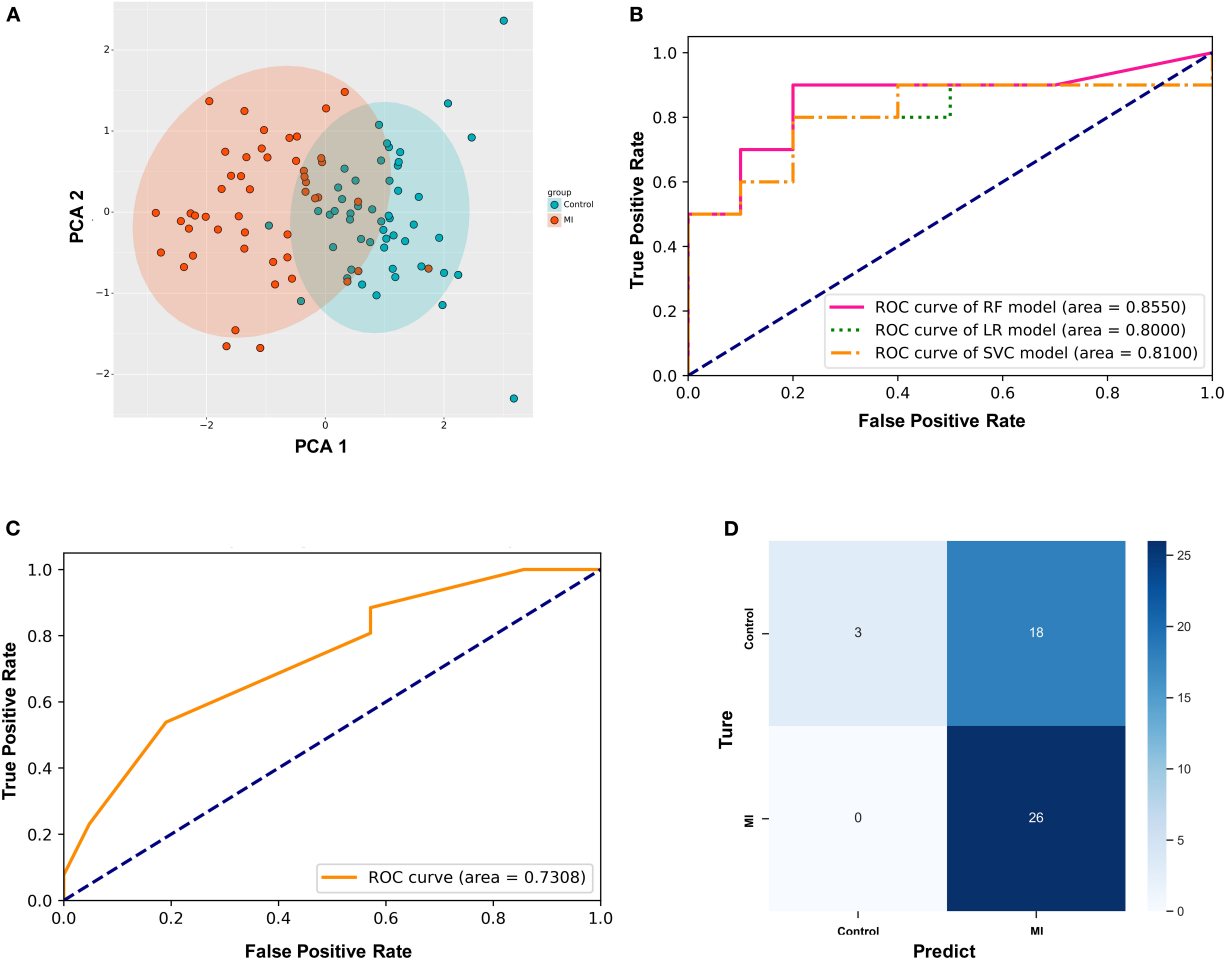
Establishment of the random forest diagnostic model based on differential ferroptosis-related genes in acute myocardial infarction (MI). **(A)** Principal component analysis (PCA) of these differentially expressed ferroptosis-related genes as dimension reduction; **(B)** the comparison of three different supervised learning models (RF, random forest; LR, logistic regression; SVC, support vector classification); **(C)** the diagnostic performance of the predictive model in the test set; **(D)** the confusion matrix of the test set.

First, PCA was performed on the above differentially expressed ferroptosis-related genes as a dimension reduction strategy. The results demonstrate that the MI and the control groups could be distinguished accurately (Figure 4A), which means that these differential genes might be treated as independent feature parameters for the diagnosis of acute MI. Then, the GSE66360 data set including 49 acute MI patients with strict diagnostic criteria and 50 healthy controls was taken as the training and validation sets (4:1), and the GSE48060 data set including 26 acute MI patients with non-recurrent events and 21 normal controls was treated as the test set.

After utilizing the K-fold cross-validation with cv = 15, the generalization ability of the training set was improved to prevent overfitting. Then, three different supervised machine learning algorithms, including logistic regression, support vector classification, and random forest, were attempted to construct the diagnostic prediction model of acute MI. The evaluating results of all three algorithms is shown in Table 2. The Kolmogorov–Smirnov (KS) values reflect the power of the binary model to classify positive and negative samples. The random forest algorithm is shown to take the leading advantage of distinguishing the two groups with KS = 0.70 in this study, and the KS values of the other two algorithms was 0.60. Admittedly, we also found that the diagnostic accuracy of the random forest model (accuracy = 0.75) was not as good as the other two models (accuracy = 0.80). However, it was far from enough to rely on accuracy to evaluate the diagnostic power, which was easily affected by the bias caused by the imbalance of categories. In other words, the recall rate was another evaluation feature that could not be ignored for diagnosing acute MI. The recall rate of the random forest algorithm (recall = 0.90) was higher than the other two models (recall = 0.80). It was indicated that the random forest model could minimize the missed cases of acute MI, which might provide a sufficient treatment window and significantly improve the prognosis of patients.

**Table 2:** Comparison of the diagnostic efficacy of three different supervised learning models.

**Model**	**Precision**	**Recall**	**F1-score**	**Accuracy**	**Error**	**KS**
Random forest	0.69	0.90	0.78	0.75	0.25	0.70
Logistic regression	0.80	0.80	0.80	0.80	0.20	0.60
Support vector classification	0.80	0.80	0.80	0.80	0.20	0.60

In addition, the ROC curves of the validation set from GSE66360 also described that the area under the curve (AUC) of the random forest model was 0.8550, which was higher than AUC = 0.80 and 0.81 of the other two groups (Figure 4B). Hence, the random forest algorithm was selected to further construct the acute MI diagnostic model after comprehensively analyzing all these parameters.

Simultaneously, GSE48060 was used as a test set to externally verify the diagnostic model based on the random forest algorithm. Figure 4D shows the ROC curve verified by external data, and its AUC is 0.7308 (Figure 4C), demonstrating a compelling diagnostic performance. What's more, the confusion matrix was visualized to evaluate the classification model (Figure 4D). Twenty-six patients with MI were correctly classified, and three healthy volunteers were identified as the control group. There was no misidentification of patients with MI as healthy people, which meant that this method could effectively reduce the false negative rate. Admittedly, we also noticed that some healthy people were misclassified as MI (*n* = 18).

## Conclusion

In this study, we first identified eight differentially expressed ferroptosis-related genes in CECs of patients with acute MI and analyzed their potential functions by means of two GEO data sets. Compared with the performance of different supervised learning models, we established a random forest diagnostic models of MI based on these ferroptosis-related genes in CECs (AUC = 0.8550) through K-fold cross-validation and verified it with another data set (AUC = 0.7308).

## Discussion

With the increase of global aging, how to deal with the medical challenges brought about by aging is a topic of general concern (40). Age-related diseases such as cardiovascular diseases, especially coronary artery diseases, continue to be a major threat to human health in the future. Advances in interventional technology and reperfusion therapy in recent years have effectively improved the prognosis of MI. However, there is still more attention that needs to be paid, and the early diagnosis of MI is still a key factor restricting mortality and prognosis.

As a kind of disease caused by insufficient myocardial perfusion, MI mainly contributes to coronary atherosclerosis. Although the current diagnostic methods are established on a series of biomarkers based on myocardial injury, which cannot give early warning when the myocardium has just appeared insufficient without yet being damaged. Not being a specific feature of MI, myocardial injury in many cardiomyopathies can also interfere with diagnosis in addition to the delayed diagnosis. Therefore, it is imperative to advance the diagnostic window of acute MI and develop diagnostic biomarkers that reflect myocardial hypoperfusion directly (9).

As a novel kind of iron-dependent programmed cell death, ferroptosis was first proposed in 2012 (41). The decrease in the activity of glutathione peroxidase (GPX4) and the depletion of glutathione interrupt the metabolic reaction of lipid oxides, which induces the Fe^2+^ to produce ROS, thereby promoting ferroptosis. Its sensitivity involves a large number of cellular metabolic processes, including amino acid, iron, and polyunsaturated fatty acid metabolism. Hence, the induction of ferroptosis leads to the increase of intracellular lipid ROS, and this regulating process could be inhibited by lipid antioxidants (17). As iron-rich and ROS production–based organelles, mitochondria are considered to be the critical place for ferroptosis with specific lipid precursors. Studies in recent years show that ferroptosis is associated with tumors (42), stroke (12), cerebral hemorrhage (43), and renal failure (44). However, the relationship between MI, especially the vascular endothelium and ferroptosis, still remains unknown.

Artificial intelligence and machine learning are important productivity tools in the twenty-first century (45). Different from traditional biomedical research, artificial intelligence, and machine learning are dedicated to learning natural laws from massive amounts of high-throughput data and then using the natural learned laws to predict unknown data, which are widely used in computer vision (46), natural language processing (47), biological features identification (48), and other fields. In the field of oncology research, a large number of machine learning diagnostic models based on gene expression have been widely developed and applied because samples of cancer can be obtained more conveniently through pathology. However, a majority of clinical prediction models are based on traditional risk factors and biomarkers for model fitting in non-tumor research fields. For example, a retrospective cohort study was used to construct a random forest model for atrial fibrillation diagnosis (49).

By means of machine learning and bioinformatics technology, our study first revealed the differential expression of ferroptosis genes in CECs of patients with acute MI. Meanwhile, all the results were verified in different data sets, which first implied that ferroptosis may be involved in regulating the metabolism of CECs in acute MI. On one hand, the eight ferroptosis-related genes described in this article have been verified and functionally confirmed through different bioinformatics technologies (GO enrichment analysis, PPI interaction analysis). Among them, CXCL2 (30), JDP2 (32), TLR4 (33), ATF3 (36), and FTH1 (39) are reported to participate in the regulation of a variety of cardiovascular diseases through different pathways, and Jun and DDIT3 were first described to be related to acute MI. All these results provide the direction and cornerstone for subsequent basic experiments to explore the role of ferroptosis regulation mechanism in the pathogenesis of acute MI.

On the other hand, this study constructed a random forest diagnostic model of acute MI through the above eight differentially expressed ferroptosis-related genes in CECs. The AUC of some clinical prediction models is extremely high, and their external verification has unsatisfactory results due to the overfitting caused by the small sample data. In this study, the K-fold cross-validation with cv = 15 was utilized to improve the generalization power. Hence, the variability of AUC between our validation and test sets was small enough to be satisfying. Meanwhile, we compared the performance of three supervised machine learning algorithms, including logistic regression, support vector classification, and random forest. After comprehensively evaluating KS, accuracy, recall, and AUC of all these three algorithms, this random forest model showed good diagnostic performance (AUC = 0.8550) and was validated in different data sets (AUC = 0.7308), which provides new ideas and directions for finding new MI-specific biomarkers in advance of the diagnosis window. What is more, the results of a confusion matrix indicate that this model has a strong ability to eliminate false negative interference, which is critical for MI with a very high fatality rate. Changes of gene expression level are the first step in the occurrence and development of diseases. The application of machine learning to analyze different gene expression levels can help explain the original mechanism of the disease and can build the first line of defense for disease diagnosis and early warning at the same time, which plays a strong guiding role of diseases such as acute MI with rapid disease development, high fatality rate, and no obvious symptoms in the early stage.

Compared with the traditional diagnostic model based on the detection of myocardial injury markers, the new model in this article based on the ferroptosis-related genes of CECs focuses more on reflecting the damage of endothelial function and the non-invasive screening for high-risk populations. Vascular endothelial injury is the key factor and initiating link of atherosclerosis. Normally, the anti-inflammatory system composed of cytokines and endothelial progenitor cells in the body repairs damaged endothelium and blood vessels. However, when the endothelial anti-inflammatory self-repair system is exhausted, endothelial cells develop a series of dysfunctions, including aging, autophagy, apoptosis, ferroptosis, etc. (50, 51), which causes endothelial cells to leave the blood vessel wall and enter circulation, which, in turn, leads to a series of undesirable consequences, such as vascular plaque formation, vascular remodeling, inflammation, vasoconstriction, thrombosis formation, and even plaque rupture. In fact, changes in CECs are important predictors of cardiovascular events before the development of atherosclerotic morphological changes (52). At present, there have been a series of research on the evaluation of CEC functions (53, 54). So far, there is no reliable and recognized gold standard for the detection of functional changes of CECs (55). The identification and integrated analysis of ferroptosis-related genes in CECs in this model are helpful to reflect the early functional changes of CECs, which is ready for judging vascular endothelial function and predicting the occurrence of cardiovascular events, especially for early non-invasive detection of high-risk population screening.

It also cannot be ignored that coronary microcirculation dysfunction, an important independent prognostic factor for cardiovascular events, is inseparably related to functional changes in endothelial cells (56), which can lead to a decrease in coronary blood flow and myocardial perfusion. However, conventional coronary angiography cannot detect microcirculation cracks. At present, the methods for evaluating microcirculation disorders are mainly based on invasive methods, such as fractional flow reserve (FFR), and imaging detection, such as cardiovascular magnetic resonance (CMR). Studies suggest that microvascular occlusion (MVO) is inseparable from the swelling of capillary endothelial cells. The lack of endothelial integrity and functionality leads to the release of large amounts of cytokines, which, in turn, activates neutrophils and platelets, contributing to the formation of microthrombi. In this pathological process, the functional changes of CECs are considered to be an indispensable link. This model based on ferroptosis-related genes in CECs can directly reflect the regulation of ferroptosis-related networks in CECs, which may help determine whether the patient is in the process of MVO and the degree of MVO. In subsequent studies, the inclusion of more data related to MVO patients, such as FFR and CMR data collection, can help enhance the model's advantages in judging microcirculation lesions, which shows a large potential value in early non-invasive and accurate identification of patients with coronary microcirculation disorders.

Meanwhile, models based on CECs not only have important clinical significance for the diagnosis of endothelial function damage, but also play a crucial role in the risk stratification and prognosis of coronary heart disease. Early research on CECs focused on changes in their quantification, and the increase of CEC counts was shown to predict cardiovascular event risk to a large extent among patients with acute coronary syndrome (57), which suggests CECs would be applied for the judgment of long-term prognosis of MI. The development of more biomarker clusters with clinical application potential based on the internal genomics, transcriptomics, and secreted cytokines of CECs is still in its infancy, which is yet an unsolved mystery waiting for people to decipher. Due to the limitation of public data sets, data on patient severity is unfortunately not included in this model. However, according to the model constructed in this article and the support of the previous literature, it is very necessary in the subsequent cohort studies to associate the severity of coronary occlusion or the incidence of long-term cardiovascular events with ferroptosis-related genes in CECs to expand the scope of application of the model.

Of course, this study also has a few shortcomings. First, the biological functions of co-expressed genes have not been further explored through weighted gene co-expression network analysis and other technologies due to the small number of differentially expressed ferroptosis-related genes. Second, this model is not compared with some traditional clinical risk models because we cannot obtain individual specific clinical data from public data sets. This undoubtedly reduces the reliability of our results. However, every coin has two sides. Since the Framingham Study (58) proposed risk stratification for coronary heart disease, various risk scoring systems for coronary heart disease have been quickly built. The GRACE (59) and TIMI (60) risk scoring systems are two relatively representative scoring systems released in recent years. Several independent clinical predictive variables were screened out and applied to divide patients with different risk levels in these scoring systems through multivariate logistic regression analysis of large-scale clinical trials. However, most of the clinical predictive variables screened out rely on epidemiological evidence instead of the pathogenesis of the disease. Therefore, these traditional models can only perform macroscopic stratification without subtly reflecting the real endothelial function status of patients, not to mention achieving the purpose of precision medicine (61). In contrast, this diagnostic model based on ferroptosis-related genes has an irreplaceable advantage in reflecting the patient's immediate endothelial function status. It can provide diagnostic prediction models with pathological progress from the perspective of genetic and molecular pathology. In fact, these two kinds of diagnostic models are absolutely not opposite, and there is no sense comparing them in a single scale. On the contrary, they are complementary to pool their experiences. On one hand, the traditional model provides a macro-level risk assessment based on the past average body state of the patients. On the other hand, the novel gene–related score provides accurate evidence such as immediate and subtle molecular pathological changes for judging the pathological process of the patients. Under this cooperative consensus, future research directions should be to integrate traditional risk and novel gene–related scores and jointly develop a new model in order to help evaluate the patients and guide treatment in the mode of precision medicine. In addition, high-quality data determines the pros and cons of machine learning. The training and validation tests were built on the data set that was matched in both gender and age in the experimental and control groups so that the interference of potential confounding factors on our model could be eliminated. However, other data sources used in this study all come from the GEO database without additional clinical features. In future studies, traditional risk factors, such as age, gender, and hypertension in the cohort could be added to the existing models to construct new combined diagnostic model tools. Meanwhile, this diagnostic model can be further compared with myocardial markers such as cTnT, or a novel diagnostic model could be constructed by these ferroptosis-related genes in CECs combined with cTnT. What is more, there are different subtypes of acute MI (62), and different subtypes need different treatment measures. For example, acute ST-segment elevation MI (STEMI) requires immediate interventional treatment, and some non-ST-segment elevation MI (NSTEMI) could choose selective intervention. Whether ferroptosis-related genes are differentially expressed in these different subtypes of MI and play different regulatory roles remains to be revealed. As is known to all, cardiovascular disease is a complex pathological process involving multiple factors and multilevel biomarkers should be established for different types or stages of the same disease to precise classification and treatment through various machine learning algorithms. In fact, the method of constructing clinical prediction models based on differentially expressed genes related to tumor incidence, metastasis, and prognosis risk via gene chip screening has been widely used in the research of different tumors (63–65). However, a model based on gene-related score in the research of cardiovascular diseases is still in its infancy due to the lack of traditional pathological specimens. Because CECs stand for changes in endothelial cell function to a large extent, enrichment and detection of differential genes may be helpful for assessing vascular endothelial dysfunction, especially identifying the risk of early coronary heart disease. Meanwhile, whether the diagnostic model based on ferroptosis-related gene labels could be practically accessible in clinical practice is worthy of attention. At present, relatively mature CEC rapid enrichment schemes have been constructed (66), and a rapid test kit based on the eight ferroptosis-related genes included in this model could be designed and produced with available test results within 4–6 h. In the next study, the cohort can be expanded to optimize and improve the model, which might reduce the missed diagnosis of early coronary heart disease and acute MI due to the heterogeneity of myocardial enzyme spectrum and the limitations of invasive coronary angiography. It is believed that integrating diverse molecular tags through machine learning can guide clinicians to more reasonable management and treatment of acute coronary syndromes in the future.

There is no doubt that the precision medicine bring about a revolution in the medical world and change the whole clinical practice. With the continuous development of artificial intelligence and machine learning, the discovery and confirmation of biomarker groups becomes possible under the maturity of massive biological information data analysis technology. Fully discovering and verifying the biomarker groups of different types and stages of MI help to comprehensively improve the prediction risk power of cardiovascular and cerebrovascular diseases, thereby reducing the mortality rate and improving the prognosis of MI.

## Data Availability Statement

The datasets presented in this study can be found in online repositories. The names of the repository/repositories and accession number(s) can be found in the article/Supplementary Material.

## Author Contributions

PJ and CY conceived the need for the article. CY finished the analysis and drafted the initial version. SJ helped revise the manuscript. PJ put forward many constructive comments for the final version. CY provided the production of the flowchart. All authors read and approved the manuscript.

## Conflict of Interest

The authors declare that the research was conducted in the absence of any commercial or financial relationships that could be construed as a potential conflict of interest.
