# Supplementary material for: Development and Validation of a Random Forest Diagnostic Model of Acute Myocardial Infarction Based on Ferroptosis-Related Genes in Circulating Endothelial Cells
Source: Front Cardiovasc Med. 2021 Jun 28;8:663509. doi: 10.3389/fcvm.2021.663509 (PMC8274450; doi:10.3389/fcvm.2021.663509)
Supplement: Supplementary file 1 [file Table_1.docx]

**Supplementary Table S1. The collected ferroptosis-related genes and their roles from from the FerrDb database and other previous literatures.**

| Gene Symbol | Role in Ferroptosis |
| --- | --- |
| RPL8 | driver |
| ATP5MC3 | driver |
| CS | driver |
| EMC2 | driver |
| NOX1 | driver |
| CYBB | driver |
| NOX3 | driver |
| NOX5 | driver |
| DUOX1 | driver |
| DUOX2 | driver |
| G6PD | driver |
| PGD | driver |
| VDAC2 | driver |
| PIK3CA | driver |
| FLT3 | driver |
| SCP2 | driver |
| TP53 | driver |
| LPCAT3 | driver |
| NRAS | driver |
| KRAS | driver |
| HRAS | driver |
| TFR2 | driver |
| GOT1 | driver |
| CARS1 | driver |
| ATG7 | driver |
| NCOA4 | driver |
| ALOX12B | driver |
| ALOX15B | driver |
| ALOXE3 | driver |
| PHKG2 | driver |
| ACO1 | driver |
| SLC38A1 | driver |
| GLS2 | driver |
| G6PDX | driver |
| ULK1 | driver |
| ATG3 | driver |
| ATG4D | driver |
| ATG5 | driver |
| BECN1 | driver |
| MAP1LC3A | driver |
| GABARAPL2 | driver |
| GABARAPL1 | driver |
| ATG16L1 | driver |
| WIPI1 | driver |
| WIPI2 | driver |
| SNX4 | driver |
| ATG13 | driver |
| ULK2 | driver |
| SAT1 | driver |
| EGFR | driver |
| MAPK3 | driver |
| MAPK1 | driver |
| BID | driver |
| ZEB1 | driver |
| KEAP1 | driver |
| DPP4 | driver |
| ALOX15 | driver |
| CDKN2A | driver |
| PEBP1 | driver |
| SOCS1 | driver |
| CDO1 | driver |
| MYB | driver |
| MAPK8 | driver |
| MAPK9 | driver |
| SLC1A5 | driver |
| CHAC1 | driver |
| MAPK14 | driver |
| LINC00472 | driver |
| PRKAA2 | driver |
| PRKAA1 | driver |
| ABCC1 | driver |
| MIR6852 | driver |
| ACVR1B | driver |
| TGFBR1 | driver |
| BAP1 | driver |
| EPAS1 | driver |
| HILPDA | driver |
| HIF1A | driver |
| ALOX12 | driver |
| ACSL4 | driver |
| HMOX1 | driver |
| IFNG | driver |
| ANO6 | driver |
| LPIN1 | driver |
| HMGB1 | driver |
| TNFAIP3 | driver |
| TLR4 | driver |
| NOX4 | driver |
| ATF3 | driver |
| ATM | driver |
| YY1AP1 | driver |
| EGLN2 | driver |
| MIOX | driver |
| TAZ | driver |
| MTDH | driver |
| IDH1 | driver |
| SIRT1 | driver |
| FBXW7 | driver |
| PANX1 | driver |
| DNAJB6 | driver |
| BACH1 | driver |
| LONP1 | driver |
| PTGS2 | marker |
| DUSP1 | marker |
| NOS2 | marker |
| NCF2 | marker |
| MT3 | marker |
| UBC | marker |
| ALB | marker |
| TXNRD1 | marker |
| SRXN1 | marker |
| GPX2 | marker |
| BNIP3 | marker |
| OXSR1 | marker |
| SELENOS | marker |
| ANGPTL7 | marker |
| DDIT4 | marker |
| LOC284561 | marker |
| ASNS | marker |
| TSC22D3 | marker |
| DDIT3 | marker |
| JDP2 | marker |
| SESN2 | marker |
| SLC1A4 | marker |
| PCK2 | marker |
| TXNIP | marker |
| VLDLR | marker |
| GPT2 | marker |
| PSAT1 | marker |
| LURAP1L | marker |
| SLC7A5 | marker |
| HERPUD1 | marker |
| XBP1 | marker |
| CBS | marker |
| ZNF419 | marker |
| KLHL24 | marker |
| TRIB3 | marker |
| ZFP69B | marker |
| ATP6V1G2 | marker |
| VEGFA | marker |
| GDF15 | marker |
| TUBE1 | marker |
| ARRDC3 | marker |
| CEBPG | marker |
| SNORA16A | marker |
| RGS4 | marker |
| BLOC1S5-TXNDC5 | marker |
| LOC390705 | marker |
| KIM-1 | marker |
| IL6 | marker |
| CXCL2 | marker |
| RELA | marker |
| HSD17B11 | marker |
| AGPAT3 | marker |
| SETD1B | marker |
| FTL | marker |
| MAFG | marker |
| IL33 | marker |
| SLC40A1 | marker |
| TF | marker |
| TFRC | marker |
| FTH1 | marker |
| HAMP | marker |
| STEAP3 | marker |
| DRD5 | marker |
| DRD4 | marker |
| MAP3K5 | marker |
| SLC2A1 | marker |
| SLC2A3 | marker |
| SLC2A6 | marker |
| SLC2A8 | marker |
| SLC2A12 | marker |
| GLUT13 | marker |
| SLC2A14 | marker |
| EIF2AK4 | marker |
| EIF2S1 | marker |
| ATF4 | marker |
| ALOX5 | marker |
| ACSF2 | marker |
| IREB2 | marker |
| GPX4 | marker |
| NFE2L2 | marker |
| ELAVL1 | marker |
| SLC3A2 | marker |
| TFAP2C | marker |
| SP1 | marker |
| HBA1 | marker |
| NNMT | marker |
| PLIN4 | marker |
| HIC1 | marker |
| STMN1 | marker |
| RRM2 | marker |
| CAPG | marker |
| HNF4A | marker |
| NGB | marker |
| YWHAE | marker |
| GABPB1 | marker |
| AURKA | marker |
| MIR4715 | marker |
| RIPK1 | marker |
| PRDX1 | marker |
| MIR30B | marker |
| SLC7A11 | suppressor |
| AKR1C1 | suppressor |
| AKR1C2 | suppressor |
| AKR1C3 | suppressor |
| RB1 | suppressor |
| HSPB1 | suppressor |
| HSF1 | suppressor |
| GCLC | suppressor |
| SQSTM1 | suppressor |
| NQO1 | suppressor |
| MUC1 | suppressor |
| MT1G | suppressor |
| CISD1 | suppressor |
| FANCD2 | suppressor |
| FTMT | suppressor |
| HELLS | suppressor |
| FADS2 | suppressor |
| SRC | suppressor |
| STAT3 | suppressor |
| PML | suppressor |
| MTOR | suppressor |
| NFS1 | suppressor |
| TP63 | suppressor |
| CDKN1A | suppressor |
| MIR137 | suppressor |
| ENPP2 | suppressor |
| FH | suppressor |
| CISD2 | suppressor |
| MIR9-1 | suppressor |
| MIR9-2 | suppressor |
| MIR9-3 | suppressor |
| ISCU | suppressor |
| ACSL3 | suppressor |
| OTUB1 | suppressor |
| CD44 | suppressor |
| LINC00336 | suppressor |
| BRD4 | suppressor |
| PRDX6 | suppressor |
| MIR17 | suppressor |
| SCD | suppressor |
| NF2 | suppressor |
| ARNTL | suppressor |
| JUN | suppressor |
| CA9 | suppressor |
| TMBIM4 | suppressor |
| HSPA5 | suppressor |
| PLIN2 | suppressor |
| MIR212 | suppressor |
| Fer1HCH | suppressor |
| AIFM2 | suppressor |
| LAMP2 | suppressor |
| ZFP36 | suppressor |
| PROM2 | suppressor |
| CHMP5 | suppressor |
| CHMP6 | suppressor |
| CAV1 | suppressor |
| GCH1 | suppressor |
